# Extracellular vesicles, RNA sequencing, and bioinformatic analyses: Challenges, solutions, and recommendations

**DOI:** 10.1002/jev2.70005

**Published:** 2024-12-03

**Authors:** Rebecca T. Miceli, Tzu‐Yi Chen, Yohei Nose, Swapnil Tichkule, Briana Brown, John F. Fullard, Marilyn D. Saulsbury, Simon O. Heyliger, Sacha Gnjatic, Natasha Kyprianou, Carlos Cordon‐Cardo, Susmita Sahoo, Emanuela Taioli, Panos Roussos, Gustavo Stolovitzky, Edgar Gonzalez‐Kozlova, Navneet Dogra

**Affiliations:** ^1^ Department of Pathology, Molecular and Cell‐Based Medicine Icahn School of Medicine at Mount Sinai New York New York USA; ^2^ Department of Immunology Icahn School of Medicine at Mount Sinai New York New York USA; ^3^ Department of Oncological Sciences Icahn School of Medicine at Mount Sinai New York New York USA; ^4^ Department of Psychiatry Icahn School of Medicine at Mount Sinai New York New York USA; ^5^ Department of Genetics and Genomics Sciences Icahn School of Medicine at Mount Sinai New York New York USA; ^6^ Center for Disease Neurogenetics, Icahn School of Medicine at Mount Sinai New York New York USA; ^7^ Friedman Brain Institute, Icahn School of Medicine at Mount Sinai New York New York USA; ^8^ Department of Pharmaceutical Sciences, School of Pharmacy Hampton University Hampton Virginia USA; ^9^ Department of Medicine Icahn School of Medicine at Mount Sinai New York New York USA; ^10^ Department of Urology Icahn School of Medicine at Mount Sinai New York New York USA; ^11^ Cardiovascular Research Institute, Icahn School of Medicine at Mount Sinai New York New York USA; ^12^ Department of Population Health and Science Icahn School of Medicine at Mount Sinai New York New York USA; ^13^ Department of Thoracic Surgery Icahn School of Medicine at Mount Sinai New York New York USA; ^14^ Center for Precision Medicine and Translational Therapeutics James J. Peters VA Medicinal Center Bronx New York USA; ^15^ Mental Illness Research Education and Clinical Center (MIRECC) James J. Peters VA Medicinal Center Bronx New York USA; ^16^ Biomedical Data Sciences Hub (Bio‐DaSH), Department of Pathology, NYU Grossman School of Medicine New York New York USA; ^17^ Icahn Genomics Institute, Icahn School of Medicine at Mount Sinai New York New York USA; ^18^ AI and Human Health Icahn School of Medicine at Mount Sinai New York New York USA

**Keywords:** bioinformatics, EVs, extracellular vesicles, long‐read sequencing, short‐read sequencing, small RNA, transcriptomics

## Abstract

Extracellular vesicles (EVs) are heterogeneous entities secreted by cells into their microenvironment and systemic circulation. Circulating EVs carry functional small RNAs and other molecular footprints from their cell of origin, and thus have evident applications in liquid biopsy, therapeutics, and intercellular communication. Yet, the complete transcriptomic landscape of EVs is poorly characterized due to critical limitations including variable protocols used for EV‐RNA extraction, quality control, cDNA library preparation, sequencing technologies, and bioinformatic analyses. Consequently, there is a gap in knowledge and the need for a standardized approach in delineating EV‐RNAs. Here, we address these gaps by describing the following points by (1) focusing on the large canopy of the EVs and particles (EVPs), which includes, but not limited to – exosomes and other large and small EVs, lipoproteins, exomeres/supermeres, mitochondrial‐derived vesicles, RNA binding proteins, and cell‐free DNA/RNA/proteins; (2) examining the potential functional roles and biogenesis of EVPs; (3) discussing various transcriptomic methods and technologies used in uncovering the cargoes of EVPs; (4) presenting a comprehensive list of RNA subtypes reported in EVPs; (5) describing different EV‐RNA databases and resources specific to EV‐RNA species; (6) reviewing established bioinformatics pipelines and novel strategies for reproducible EV transcriptomics analyses; (7) emphasizing the significant need for a gold standard approach in identifying EV‐RNAs across studies; (8) and finally, we highlight current challenges, discuss possible solutions, and present recommendations for robust and reproducible analyses of EVP‐associated small RNAs. Overall, we seek to provide clarity on the transcriptomics landscape, sequencing technologies, and bioinformatic analyses of EVP‐RNAs. Detailed portrayal of the current state of EVP transcriptomics will lead to a better understanding of how the RNA cargo of EVPs can be used in modern and targeted diagnostics and therapeutics. For the inclusion of different particles discussed in this article, we use the terms large/small EVs, non‐vesicular extracellular particles (NVEPs), EPs and EVPs as defined in MISEV guidelines by the International Society of Extracellular Vesicles (ISEV).

## Graphical abstract

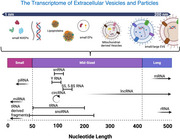

## 1 INTRODUCTION

All cells secrete heterogeneous micro‐/nano‐scaled extracellular vesicles (EVs) as part of regular homoeostasis, intercellular communication and cargo disposal (Dalton, 1975; Krishn et al., 2020; Soleymani et al., 2023; Tkach & Théry, 2016; Trams et al., 1981; Valadi et al., 2007). EVs are in blood, urine, cerebrospinal fluid, cell culture media and a variety of other fluids (Chen et al., 2022; Lucien et al., 2023; Sandau et al., 2024; Théry et al., 2018). Recent evidence shows that EVs encapsulate small (∼15–200 nucleotides (nt)) functional RNAs (Bellingham et al., 2012; Crescitelli et al., 2021; Driedonks & Nolte‐T'Hoen, 2019; Lässer et al., 2011; Skog et al., 2008; Valadi et al., 2007), which can serve as extracellular messengers and induce recipient cells to change their behaviour (Alem et al., 2021; Krishn et al., 2020; Lässer et al., 2011; Morello et al., 2013; Sharma, 2014; Valadi et al., 2007). Consequently, EVs and their RNA molecules are being recognized as a ‘messenger of the cell’ (Bellingham et al., 2012; Eldh et al., 2012; Kalluri & LeBleu, 2020; Valadi et al., 2007). These findings have resulted in major large‐scale transcriptomic efforts (such as the NIH‐funded extracellular RNA communication [exRNA] consortium [ERCC]) to delineate exRNA from various cells, disease types and biofluids (Everaert et al., 2019; Lucien et al., 2023; Murillo et al., 2019; Rozowsky et al., 2019; Srinivasan et al., 2019; Srinivasan et al., 2019). Recent literature has shown that EVPs carry distinct small RNA transcriptional cargo from their cell of origin (Chen et al., 2022), including miRNA (Bellingham et al., 2012; Chand et al., 2021; Valadi et al., 2007), tRNA (Chen et al., 2022; Shurtleff et al., 2017), circular RNA (Li et al., 2015), long non‐coding RNA (Wei et al., 2017), Y RNA (Chen et al., 2022; Shurtleff et al., 2017), fragmented or whole messenger RNA (Wei et al., 2017) and unannotated dark matter RNA (Dogra et al. 2024). Subsequently, a large subset of EV‐associated small RNAs have been reported as potential biomarkers (Chen et al., 2022; Chen et al., 2023; Gaglani et al., 2021; Ho et al., 2022; Urabe et al., 2020), therapeutic mediators (Gaglani et al., 2021; Lässer et al., 2011; Li et al., 2023) and post‐translational regulators of gene expression (Aday et al., 2021; Alem et al., 2021; Chen et al., 2018; Gaglani et al., 2021; Khadka et al., 2023; Li et al., 2023).

**FIGURE 1:**
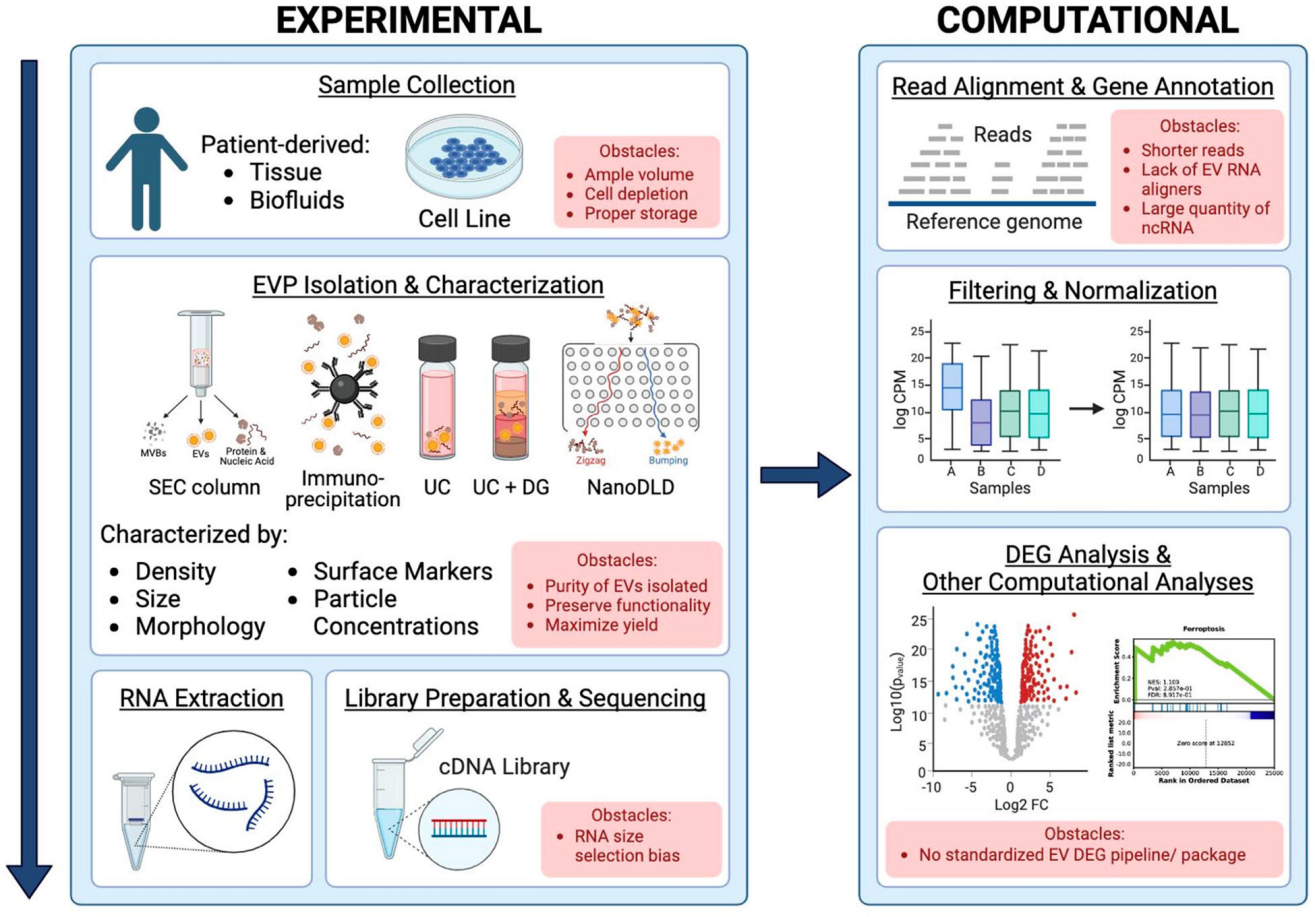
A summary of experimental and computation tools used for EV and RNA isolation and transcriptomic analysis of small RNAs from extracellular vesicles and particles. Current obstacles within each section are highlighted in red and discussed in Section 8. DG, density gradient; nanoDLD, nanofluidic deterministic lateral displacement; SEC, size exclusion chromatography; UC, ultracentrifugation.

Recent advances in EV isolation and sequencing technologies have enhanced our ability to investigate the role of extracellular transcriptomes in fundamental biology, extracellular communication and liquid biopsy‐based diagnostics (Athota et al., 2023; Reátegui et al., 2018; Smith et al., 2018; Wei et al., 2017). Importantly, with major advances in next‐generation sequencing technologies and novel computational tools (Figure 1), it is now possible to study the complete transcriptome, providing an unparalleled opportunity to assess the largely uncharacterized EV‐RNA subtypes (Abrams et al., 2019; Joo et al., 2019; Lu et al., 2018). However, current sequencing technologies and protocols are tailored to cellular RNAs and fail to accurately process and extract meaningful information from EV‐RNAs (Murillo et al., 2019; Rozowsky et al., 2019). Recent studies have identified critical limitations, including variable protocols used for EV‐RNA extraction, quality control, cDNA library preparation, sequencing technologies and bioinformatic analyses (suboptimal alignment of short reads (20–50 nt), a high number of multimapping reads, missing genomic annotation) and poor molecular cargo understanding (Chen et al., 2022; Cheng & Hill, 2022; Hoshino et al., 2020; Murillo et al., 2019; Santos & Almeida, 2021; Tesovnik et al., 2021; Vasconcelos et al., 2019; Wei et al., 2017). To date, there is a gap in knowledge for a gold standard approach to identifying small RNAs associated with EVs for better cross‐comparison between studies (Murillo et al., 2019; Srinivasan et al., 2019). To address these challenges, novel approaches and extensive benchmarking of current technologies are needed, along with the urgent development of robust, reproducible, open‐access and user‐friendly methods for EV diagnostics, quality control, and analysis.

In this review article, we seek to provide clarity on the transcriptomics landscape of EVs through an in‐depth analysis of EV biogenesis, their small RNAs landscape and the use of several transcriptomics technologies, including single‐EV omics. Notably, we not only focus on exosomes (a subset of small EVs) but include a large canopy of the extracellular vesicles and particles (EVPs), which includes, but is not limited to – large EVs (Antonyak et al., 2011; Keerthikumar et al., 2015; Kyprianou et al., 1990), small EVs (Soleymani et al., 2023; Trams et al., 1981), lipoproteins (HDL, LDL, VLDLs, ILDL and chylomicrons) (Hirowatari et al., 2008; Lutomski et al., 2018; Schneider et al., 1982), exomeres/supermeres (Zhang et al., 2018; Zhang et al., 2021), mitochondrial‐derived vesicles (Miller et al., 2022; Popov, 2022), RNA binding proteins/ribonucleic proteins (Boivin et al., 2019; Driedonks & Nolte‐T'Hoen, 2019; Jeon et al., 2022; Ripin & Parker, 2023; Schieweck et al., 2018; Wei et al., 2017) and cell‐free DNA/RNA/proteins (Murillo et al., 2019; Vagner et al., 2018; Vorperian et al., 2022). Finally, we seek to further understand the novel mechanisms and distinct cargo loading with these EVPs, and to enhance their uses as therapeutic delivery vehicles, and in liquid‐biopsy biomarker applications. Overall, we identify current challenges and provide recommendations for more cohesive RNA transcriptomics of EVPs for future studies (Dogra et al., 2021). We hope that through detailed descriptions of the current state of the art in EVP transcriptomics, we will better understand how the RNA cargo of small EVPs can be used across studies in modern and targeted diagnostics and therapeutics. Finally, our previous work on EVs (Chen et al., 2022; Chen et al., 2023; Murillo et al., 2019; Smith et al., 2018; Soleymani et al., 2023; Von Felden et al., 2022) and their transcriptomic analyses have laid the foundation of the presented work in this manuscript.

## 2 CANOPY OF DIVERSE EXTRACELLULAR VESICLES AND PARTICLES

**FIGURE 2:**
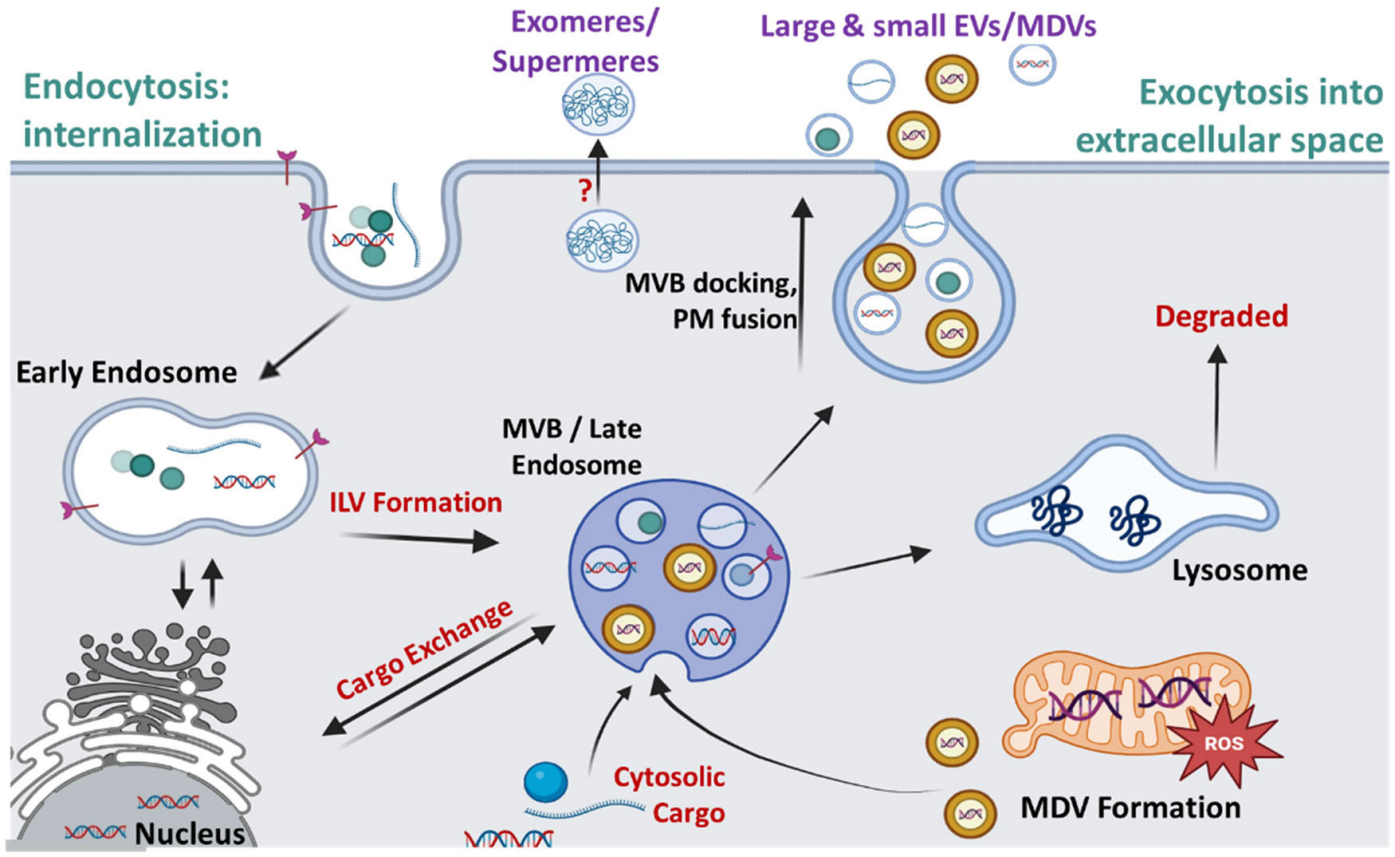
The biogenesis of various EVP subtypes, including exosomes, exomeres, supermeres and mitochondrial‐derived vesicles. Multivesicular bodies (MVB) are specialized ‘late’ endosomes derived from the plasma membrane that are sorted for lysosomal degradation or recycling via the Golgi apparatus. Intraluminal vesicles (ILVs) are formed when the membrane of the MVBs folds inward towards the lumen and leads to the budding of vesicles into the MVB lumen. ILVs may be transported to the plasma membranes and released extracellularly as exosomes. Mitochondria‐derived vesicles (MDVs) are formed under normal physiological or stress‐related conditions. During oxidative stress, the mitochondria may shed oxidized membrane proteins or damaged proteins through the budding off the damaged segments of the mitochondrial membrane for eventual degradation. Exomeres and supermeres are small (<50 nm) non‐membranous particles containing lipids, nucleic acids and proteins that are co‐isolated with small EVs from unknown biogenesis pathways.

The nomenclature and classification of EVPs (Table 1) are still under intense debate, and even the purest vesicle isolates can remain heterogeneous by current purification techniques (Théry et al., 2018; Welsh et al., 2024). The recent MISEV 2023 guidelines have reported quick‐reference EV nomenclature‐related terms (Welsh et al., 2024). Presently, a broad consensus classifies EVPs into three main classes based on the differences in their size, formation mechanism and content (Jeppesen et al., 2019; Royo et al., 2020; Théry et al., 2018; Witwer et al., 2013). Herein, we briefly discuss each EVP subtype, their biogenesis (Figure 2) and reported identification markers (Table 1). Finally, for the purpose of inclusion of different particles, we have used the terms EVs, EVPs and NVEPs as defined by the International Society of Extracellular Vesicles (ISEV) (Théry et al., 2018; Welsh et al., 2024).

**TABLE 1:** A comparison of molecular weight (kDa); zeta potential (mV); density (g/mL); diameter (nm); commonly reported markers and encapsulated RNAs for large EVs, small EVs, small EPs), very‐low‐density lipoproteins (VLDLs), low‐density lipoproteins (LDLs) and high‐density lipoproteins (HDLs).

	Large EVs		Small EVs		Small EPs			
	Reported as: ectosomes, microvesicles, large EVs, etc.	Reported as: apoptotic bodies, large EVs, etc.	Reported as: exosomes, small EVs, etc.	Reported as: mitochondrial‐derived vesicles	Exomeres	Supermeres	Midbody particles	Lipoproteins
Mol. weight (kDa)	>5*10^7^	Not reported	>5*10^5^	Not reported	Not reported	Not reported	Not reported	50–8*10^4^
Zeta potential (mV)	−25 to −30	Not reported	−15 ± 1.3	Not reported				
	Not reported	Not reported	Not reported	−10 to −27				
Density (g/mL)	>1.2	∼1.2	∼1.1	Not reported	∼1.2	Not reported	∼1.3	∼1 (VLDL), ∼1 (LDL), ∼1.1 (HDL)
Diameter (nm)	50–2000	800– 5000	∼30–200	70–150	∼40–70	<50	200–600	5–90
Reported markers	Actin, Gapdh, PKM, HSP, KIF23, RACGAO, CSE1L, ARF6, TSP, C3b and EMMPRIN	Annexin V positivity, phosphatidyl serine, Calnexin (CANX), Grp94 (HSP90B1), GM130 (GOLGA2), cytochrome C (CYC1), TOMM20, thrombospondin (TSP) and C3b	CD9/63/81, ALIX, TSG101, RAB, clathrin, flotillin, actin, HSC70, Hsp90, Hsp20, integrins, moesin, beta‐2‐microglobulin	TOMM20, ATP5A, MTCOI, NDUFB8, PDH, encapsulated mtDNA	CD9/63/81, HSPA8/1A, HSP90AB1, ACT, TUB, GAPDH,	TGFBI, VPS35/29/26A, HSPA13, and HSP90	CRIK, prominin‐1, α‐tubulin, MKLP1 and RACGAP1	Apo‐B_100_ CI, CII, AII,
Encapsulated RNAs	miRNA, mRNA, lncRNA, dsRNA	rRNAs, mtRNA	mRNAs, miRNAs	mtDNA	mRNA, miRNAs	exRNA, miR‐1246	mRNA	exRNA
References	Théry et al. (2018), Morello et al. (2013), Keerthikumar et al. (2015), Antonyak et al. (2011), Welsh et al. (2024), Raposo and Stoorvogel. (2013), Akers et al. (2013), Colombo et al. (2014), Muralidharan‐Chari et al. (2010), Abels and Breakefield (2016)	Théry et al. (2018), Welsh et al. (2024), Akers et al. (2013), Colombo et al. (2014), Pavlyukov et al. (2018), Sork et al. (2018)	Soleymani et al. (2023), Tkach and Théry. (2016), Théry et al. (2018), Urabe et al. (2020), Welsh et al. (2024), Veziroglu and Mias. (2020), Zhang et al. (2020), Abramowicz and Story (2020), Kostyushev et al. (2020), Vader et al. (2016)	Théry et al. (2018), Popov. (2022), Miller et al. (2022), Welsh et al. (2024), Picca et al. (2023), D'Acunzo et al. (2021), Heyn et al. (2023), Guan et al. (2021)	Soleymani et al. (2023), Zhang et al. (2018), Zhang et al. (2021), Jeppesen et al. (2019), Royo et al. (2020), Zhang et al. (2019), Zhang et al. (2023)	Soleymani et al. (2023), Zhang et al. (2018), Zhang et al. (2021), Zhang et al. (2023)	Rai et al. (2021), Dubreuil et al. (2007), Arai et al. (2015), Park et al. (2023), Farmer et al. (2023)	Soleymani et al. (2023), Schneider et al. (1982), Hirowatari et al. (2008), Lutomski et al. (2018), Feingold (2024), Liangsupree et al. (2021)

Large EVs are primarily characterized as plasma membrane (PM)‐derived vesicles formed by the outward budding of the PM (Kalluri & LeBleu, 2020; Raposo & Stoorvogel, 2013). Such EVs are broadly defined as >200 nm in diameter and enriched with PM markers. Frequently used large vesicle markers necessary for the differentiation of large EVs from small EVs and particles are listed in Table 1. Apoptotic bodies typically carry nuclear fragments and cellular organelles, often from damaged or dying cells (Akers et al., 2013; Théry et al., 2018; Yu et al., 2023). Typical markers for apoptotic bodies are included in Table 1.

**FIGURE 3:**
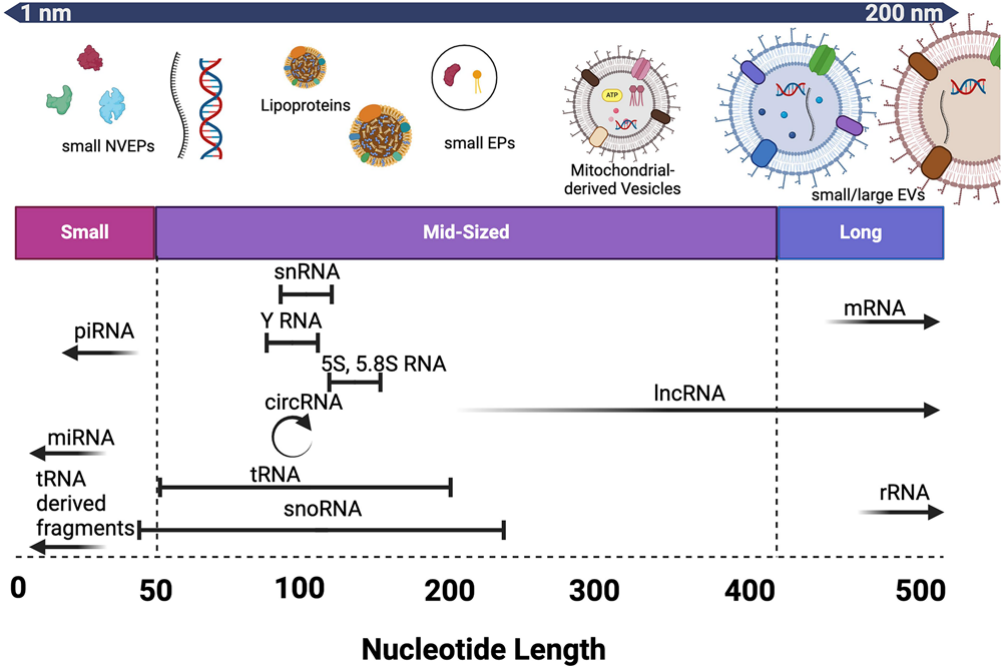
Overview of the RNA landscape of EVPs. Most commonly studied small EVP subtypes ranging from a diameter of ∼1 nm to >200 nm (top), and the most common types of RNA found within EVPs ranging from miRNA, piRNA, tRNA, snRNA, YRNA, circRNA, snoRNA, lncRNAs, mRNA and rRNA (bottom).

Small EVs biogenesis has been observed via two mechanisms: (a) an endosome fusion with PM (Pegtel & Gould, 2019), and (b) via the outward budding of the PM (Raposo & Stoorvogel, 2013). Small EVs are generally classified as ∼50–200 nm in diameter. Exosomes are a very commonly studied EVP (Fevrier et al., 2004; Johnstone et al., 1987; Trams et al., 1981). First described in the 1980s (Pan & Johnstone, 1983; Trams et al., 1981), exosomes are defined as small (∼30–200 nm) (Table 1 and Figure 3), cup‐shaped vesicles generated through the formation of multivesicular bodies (MVBs), which contain intraluminal vesicles (ILVs) (Trams et al., 1981; Johnstone et al., 1987) (Figure 2). As an MVB fuses with the PM, ILVs are released as exosomes into the extracellular space by exocytosis (Soleymani et al., 2023; Kalluri & LeBleu, 2020; Akers et al., 2013). Exosome cargo loading is initiated by the endosomal sorting complex (ESCRT), beginning in the early endosome, and continuing into the late endosome which contains ILVs marked with ubiquitinylated cargoes (Vietri et al., 2020; Kowal et al., 2014). Common cargoes include RNAs, DNAs, lipids and proteins packaged from the host cell (Valadi et al., 2007; Soleymani et al., 2023; Skog et al., 2008; Yu et al., 2022; Ludwig et al., 2019; Simpson et al., 2009). Typical exosome markers are defined in Table 1 (Théry et al., 2018; Popov, 2022; Welsh et al., 2024; Veziroglu & Mias, 2020; Colombo et al., 2014; Picca et al., 2023). Recently, small mitochondrial‐derived vesicles (70–150 nm) (Table 1) have been identified as derivatives from basal or stress‐related mitochondrial origin (Figure 2) (Popov, 2022; Miller et al., 2022; Picca et al., 2023; D'Acunzo et al., 2021; Heyn et al., 2023). Additionally, mitochondrial‐derived vesicles with cargoes from the outer membrane (lipopolysaccharides, peptidoglycans and cytosol) (Kaparakis‐Liaskos & Ferrero, 2015) and inner membrane (DNA, cytoplasmic and inner membrane proteins, and ATP) (Perez‐Cruz et al., 2013; Perez‐Cruz et al., 2015) constituents have been identified. Typical markers for mitochondrial‐derived vesicles are included in Table 1.

Finally, small (sub‐50 nm) non‐vesicular extracellular particles (NVEPs) are a subset of EVPs that have gained much attention recently (Zhang et al., 2021; Zhang et al., 2023; Rai et al., 2021), mainly due to the unique cargo associated with them and the novel isolation technologies used to isolate such small particles (Zhang et al., 2019; Zhang et al., 2023). Such examples include exomeres (Zhang et al., 2023), supermeres (Zhang et al., 2021) and midbody particles (Rai et al., 2021). Exomeres (Zhang et al., 2023) and supermeres (Zhang et al., 2021) were recently discovered and are disqualified from EV‐status due to the lack of a lipid bilayer (Théry et al., 2018). Instead, exomeres are small (<50 nm) non‐membranous particles containing lipids, nucleic acids and proteins that are co‐isolated with small EVs from unknown biogenesis pathways (Figure 2) (Soleymani et al., 2023; Zhang et al., 2019). The function and biogenesis of exomeres, supermeres and midbody particles are largely unknown and future studies are necessary to further define this EVP sub‐type (Zhang et al., 2019; Zhang et al., 2023; Rai et al., 2021).

In the 1960s, the more heavily studied non‐membrane‐bound particles called ribonucleoproteins (RNPs) or RNP granules were discovered (Rendi & Hultin, 1960). RNPs are membrane‐less intracellular RNA‐protein assemblies found in cells, including the nucleolus (Ripin & Parker, 2023). RNPs have been linked to the loading processes of RNA into EVs, including argonaute proteins (Jeppesen et al., 2019). Argonaute proteins are a key component of the miRNA‐induced silencing complex found in almost all eukaryotes, as well as some bacteria and archaea (Jeppesen et al., 2019; Hutvagner & Simard, 2008). Argonaute proteins are also associated with maintaining genome integrity, controlling protein synthesis and maintaining RNA stability (Hutvagner & Simard, 2008). Regarding their involvement with EVPs, they may also be responsible for circulating miRNA as co‐isolated fractions with exosomes (Jeppesen et al., 2019).

Another class of small particles often co‐isolated with EVs are lipoproteins (high‐density [HDL], low‐density [LDL], intermediate‐density [IDL], and very‐low‐density [VLDL] lipoproteins), ranging in size from 5 to 90 nm in diameter (Liangsupree et al., 2021). Lipoproteins are synthesized through the endoplasmic reticulum and contain distinct cargos, including RNA and cholesterol (Soleymani et al., 2023; Liangsupree et al., 2021; Claude, 1970). Lipoproteins are micelle‐like assemblies that consist of phosphatidylcholine and sphingomyelin, as well as cholesterol, fatty acids and apolipoprotein A, B and C (Hirowatari et al., 2008; Lutomski et al., 2018; Claude, 1970). In addition to lipoproteins, cell‐free nucleic acids are covered within this review (Timagiorgis et al., 2011). Cell‐free nucleic acids are found freely in biological fluids and have uses in liquid‐biopsy technologies (Timagiorgis et al., 2011). The biogenesis and function of cell‐free nucleic acids remain unknown; however, changes in concentration and quality of cell‐free DNAs/RNAs have been found to differ in healthy/diseased state patients (Pös et al., 2018). Understanding and deciphering the nuances of cell‐free nucleic acids is important for their future potential for clinical use in diagnostics.

The EVPs mentioned in this article can be isolated through a variety of well‐established protocols (see Figure 1), which are not the focus of this current review. Methods including differential ultracentrifugation (Crescitelli et al., 2021; Théry et al., 2006; Cvjetkovic et al., 2014), density gradient ultracentrifugation (Théry et al., 2006), size exclusion chromatography (SEC) (Böing et al., 2014; Gámez‐Valero et al., 2016), immunoprecipitation (Théry et al., 2006; Tauro et al., 2012), field‐flow fractionation (Zhang et al., 2018), nanofluidic deterministic lateral displacement (Smith et al., 2018; Wang et al., 2022), heat shock protein affinity (Ghosh et al., 2014) and acoustic trapping technology (Bryl‐Górecka et al., 2018; Rezel et al., 2016). Additionally, EVP characterization has been outlined by MISEV 2023 (Théry et al., 2018; Welsh et al., 2024), and can be evaluated via particle number concentration, particle size, quantification of total protein, lipids and RNA, as well as characterization of EV morphology, and by protein marker composition, as listed in Table 1. EVP characterization is necessary to deconvolute large/small EVs from other subtypes as listed in Table 1.

## 3 RNA TYPES FOUND IN EVPS

EVPs are known to encapsulate many different types of RNAs, including long (Kim et al., 2017; Prieto‐Vila et al., 2021), short (Sork et al., 2018), coding (Batagov & Kurochkin, 2013; Chiang et al., 2023) and non‐coding (Kim et al., 2017; Gezer et al., 2014; Mohankumar & Patel, 2016) RNAs (Figure 3). There is a current challenge in the field of EVP omics that includes identifying each of the mentioned RNAs below.

### 3.1 Messenger RNAs (mRNAs)

mRNAs function as protein‐coding templates that are synthesized in the nucleus and exported to the cytosol for translation (Choi et al., 2013; Lowe et al., 2017; Gomez et al., 2018). Mature post‐splicing mRNAs contain three segments, a 5′ methyl‐7‐guanosine cap, 5′ untranslated region, a coding region, a 3′ untranslated region, and a 3′ poly‐A tail (Kim et al., 2017). Full‐length and fragmented mRNA have been discovered in EVPs (Kim et al., 2017; Prieto‐Vila et al., 2021). mRNAs derived from hepatocellular carcinoma cell EVPs contained fragments ranging from 25 to 4000 nt, whereas cellular mRNAs are typically 400–12,000 nt (Prieto‐Vila et al., 2021). Currently, there is not a clear understanding of how fragmented mRNAs function in cellular communication; however, it has been theorized that EVPs are enriched in mRNAs that may code for very short proteins (Batagov & Kurochkin, 2013; Chiang et al., 2023). Additionally, it has been shown that upon delivery, fragmented mRNAs from EVs produce proteins with differing functions from their parental source (Batagov & Kurochkin, 2013; Chiang et al., 2023). Further studies are necessary to understand how full‐length and fragmented mRNAs function in the EVP space and mediate cellular communication.

### 3.2 MicroRNAs (miRNAs)

MiRNAs are small and non‐coding RNAs (∼18–22 nt), that are highly biologically stable both inside and outside cells (Boivin et al., 2019; Kim et al., 2017; Xu et al., 2022). MiRNAs can bind to mRNA to control protein expression and have unique profiles when comparing EVP isolation from diseased or normal organisms – thus giving them promise as novel biomarkers for liquid biopsy (Boivin et al., 2019; Kim et al., 2017; Xu et al., 2022). Due to their roles in modulating gene expression, miRNAs may also be useful in development, cell proliferation and differentiation, apoptosis and immune regulation, among other biological processes (Kim et al., 2017). Levels of miRNAs from EVs originating in tumour patients were reported to be significantly higher than those in healthy controls, indeed numerous miRNAs have been identified as disease‐related diagnostic and prognostic markers for hepatocellular carcinoma (Von Felden et al., 2022), breast cancer (Cha et al., 2015), prostate cancer (Morello et al., 2013) and lung adenocarcinoma (Xu et al., 2022). The sorting mechanism of miRNA into EVPs is not understood (Sork et al., 2018). However, Shurtleff et al. suggest that the RNA‐binding protein YBX1 may play a key role in sorting and selecting miRNAs into exosomes, as well as other small RNAs such as ncRNA, tRNA and Y RNA (Shurtleff et al., 2017).

### 3.3 Long non‐coding RNAs (lncRNAs)

lncRNAs were discovered in the 1970s by Berget et al. (1977). They typically consist of >200 nt and are produced from intergenic regions (Boivin et al., 2019; Kim et al., 2017; Zhang et al., 2013; St.Laurent et al., 2015). LncRNA is biologically involved in controlling many different cellular processes, including gene transcription, mRNA production, protein translation, cellular senescence and assembly of macromolecular structures (Kim et al., 2017; St.Laurent et al., 2015). LncRNAs have been proposed as potential biomarkers for cancer diagnoses, as multiple specific lncRNAs are upregulated in circulating EVPs from liver, breast and cervical cancers (Kim et al., 2017; Gezer et al., 2014; Mohankumar & Patel, 2016). In these studies, lncRNAs from EVPs had distinctly differing expression patterns compared to cells (Kim et al., 2017; Gezer et al., 2014; Mohankumar & Patel, 2016). Another proposal for the use of lncRNAs in EVPs is that they may provide mechanisms for loading miRNAs into EVs, due to significant complementary motifs between regions of lncRNAs and specific miRNAs found in prostate cancer (Ahadi et al., 2016).

### 3.4 Y RNAs

Y RNAs were discovered by Lerner et al. (1981). They are classified as a mid‐sized, non‐coding RNA (∼100 nt) that are involved with RNP complexes and DNA replication through interactions with chromatin and initiation proteins (Valkov & Das, 2020). Y RNAs are largely conserved (Gulìa et al., 2020). They also have a unique structure, with a complex three‐dimensional rigid stem and single‐stranded flexible loop (Gulìa et al., 2020). They are associated with major targets for autoimmune responses and may be useful in tumour characterization (Chen et al., 2022; Driedonks & Nolte‐T'Hoen, 2019; Gulìa et al., 2020). Y RNAs have the potential as biomarkers or molecular targets for human cancers, as they have been isolated from EVPs in paediatric brain cancer (Magaña et al., 2022) and many different solid tumours such as lung, kidney, bladder, prostate, colon and cervix (Gulìa et al., 2020). Y RNAs have been detected in EVs from multiple human cell lines and biofluids and may be involved in immune‐related processes, immune response and the establishment of a tumour microenvironment – as well as changes in the concentrations of Y RNAs in plasma EVs being associated with disease progression (Driedonks & Nolte‐T'Hoen, 2019). Additional research is necessary to further understand the roles of Y RNAs in humans, and their involvement as EVP cargoes.

### 3.5 Circular RNAs (CircRNAs)

CircRNAs are a class of non‐coding RNAs that are detected in EVPs and generated through head‐to‐tail backsplicing (Kim et al., 2017). Their lack of terminal ends increases their half‐life and protects them against degradation by exonucleases, this may enable critical translational and posttranslational functions to persevere (Kim et al., 2017). Additionally, circRNAs have been identified as sponges for some miRNAs, indicating they may be involved in the regulation and stability of mRNAs (Kim et al., 2017; Zhang et al., 2013; St.Laurent et al., 2015). CircRNAs fall into three subclasses, intronic circRNAs and exon‐intro circRNAs which are mainly found in the nucleus, whereas the exonic circRNA is typically found in both the cytoplasm and the nucleus (Han et al., 2022). In patients with pancreatic cancer, a total of 453 significantly differentially expressed circRNAs were identified, where pathway analyses indicated involvement with cellular senescence, endocytosis and cell cycle progression, among others (Li et al., 2019). Another study by Li et al. reported that circRNAs isolated from serum EVs were able to distinguish healthy patients from those with colon cancer (Li et al., 2015). Additionally, circRNAs have been identified as potential biomarkers for diseases such as schizophrenia (Guo et al., 2023).

### 3.6 Small nucleolar RNAs (snoRNAs)

snoRNAs are intron‐encoded non‐coding RNAs found in the nucleoli, consisting of 60–300 nt in length (Bellingham et al., 2012; Cammarata et al., 2022). They are responsible for post‐translational modifications and maturations of rRNAs, snRNAs and other non‐coding RNAs (Cammarata et al., 2022). They are also diverse, known to regulate cardiac‐relevant and oxidative pathways, as well as gene expression and intercellular communication (Chabronova et al., 2024). They are also enriched in EVPs compared to source cells and function to guide the chemical modification of other RNA species (O'Brien et al., 2020). SnoRNAs have been found to be upregulated in lung cancer, and are in use as predictive measures for lung cancer progression through early stages (Cammarata et al., 2022). SnoRNAs can be difficult to detect using certain RNA isolation protocols and bioinformatics pipelines as they are short in length, have unique secondary structures and lack a poly(A) tail (Chabronova et al., 2024). Further studies are necessary to identify and determine the functionality of snoRNAs in EVs.

### 3.7 Small nuclear RNA (snRNA)

snRNAs are another class of mid‐sized RNAs (∼100 nt) and are required for pre‐mRNA splicing, as well as playing a role in the regulation of RNA expression and stability through splicing‐associated processes (Boivin et al., 2019). snRNAs are found abundantly in EVs, including U6 and U1 snRNAs, which have been proposed to qualify as candidate housekeeping EV‐RNAs due to their abundance and consistency in EVs (Mosbach et al., 2021). snRNAs have also been proposed as potential biomarkers due to their differences in abundance comparing diseased vs healthy states (Li et al., 2014). One study by Kaur et al. determined that only 4% of small RNA reads aligned with snRNA in human pluripotent stem cell EVs (Kaur et al., 2018). Another study by Valkov et al. used snRNA‐seq to profile the role of snRNA isolated from EVs using murine red blood cells where results indicated that there was an enrichment of genes associated with intercellular signalling and immune response (Valkov et al., 2021).

### 3.8 Transfer RNAs (tRNAs)

tRNAs are highly structured and expressed mid‐sized (∼50–200 nt) RNA subtypes involved in the process of translation (Boivin et al., 2019). tRNAs are transcribed in the nucleus from the tRNA gene using RNA polymerase III (Weng et al., 2022). Mature full‐length tRNAs are 70–90 nt folded into an L‐shaped tertiary structure (Weng et al., 2022). In the space of the unannotated transcriptome, fragments and derivatives of tRNA have been discovered through advances in sequencing technologies (Boivin et al., 2019). Fragments derived from tRNAs are abundant species in EVs (Sork et al., 2018).

### 3.9 Ribosomal RNAs (rRNAs)

rRNAs are a major component of the ability of the ribosome to complete translation (Wei et al., 2017; Sork et al., 2018). Typically associated with the endoplasmic reticulum, rRNAs can also be found in the cytoplasm (Abramowicz & Story, 2020). The loading of rRNAs into EVs is currently controversial, where some studies indicate that up to 80% of EV cargo is identified as rRNA, whereas some studies indicate that no rRNA is discovered (Sork et al., 2018; Abramowicz & Story, 2020). Additionally, rRNA has not been shown as a potential biomarker for disease, and no specific function for rRNA loading has been identified in EVs (Abramowicz & Story, 2020). Additionally, bacterial‐EV‐derived 16S rRNA was detected in the urine of pregnant and non‐pregnant women, indicating that bacterial‐derived EVs may be secreted from gut leakage (Ricci et al., 2020).

### 3.10 Functionality of RNA in EVs

**FIGURE 4:**
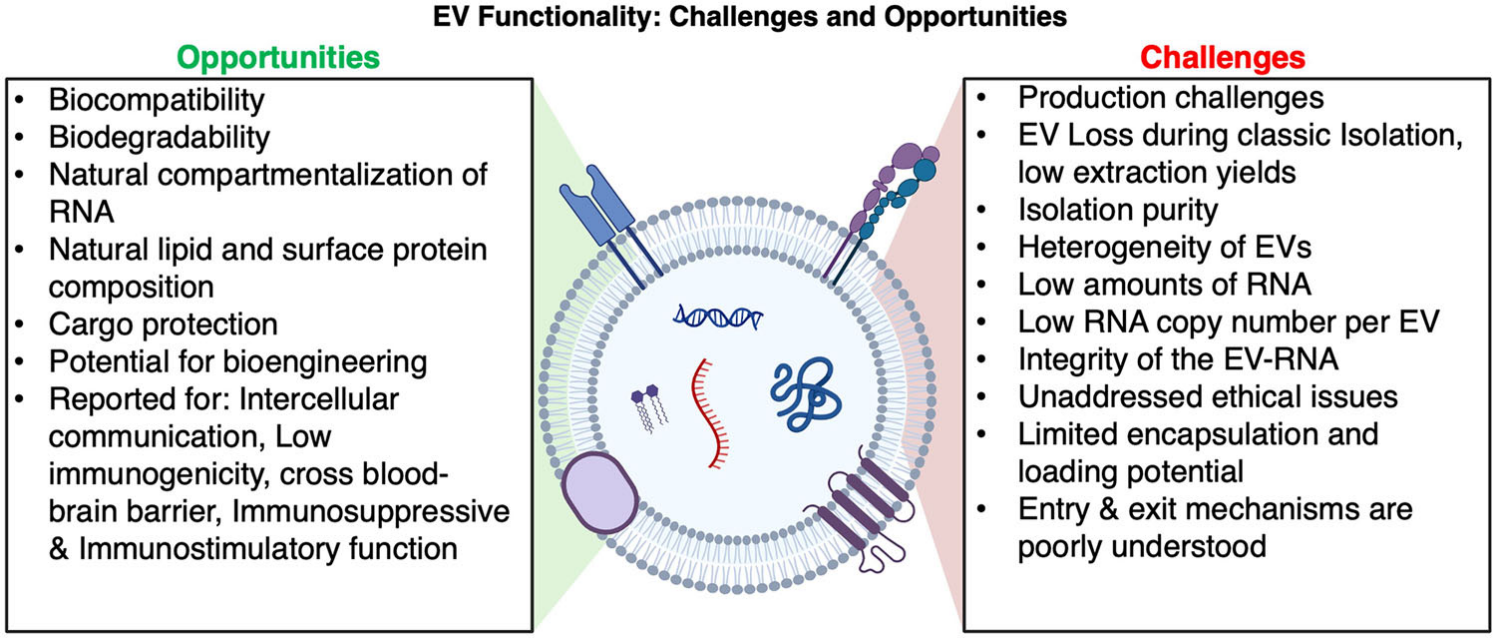
EV functionality: challenges and opportunities. Current challenges and potential opportunities with EV‐RNA functionality.

The specific functionality of RNA cargoes in EVs remains largely unknown. Some functional studies have been completed recently to investigate how EVs carrying mRNA cargoes may be translated to recipient cells (Valadi et al., 2007; Prieto‐Vila et al., 2021; Guo et al., 2022). Studies have been completed on coding RNAs isolated from EVs found in plasma that may be translated by host cells to promote the repair of cardiac cells (Danielson et al., 2018), contribute to neurodegeneration (Varcianna et al., 2019) or create favourable microenvironments for cancer progression (Abels et al., 2019) or metastasis (O'Brien et al., 2020; Hoshino et al., 2015) (Figure 4). Since our understanding of non‐coding RNAs largely remains unknown, their roles in intercellular communication as cargoes of EVs require further research (O'Brien et al., 2020). EVs also have a proposed functional role in maintaining cellular homoeostasis through the disposal of unwanted cellular materials, such as misfolded proteins, removal of cholesterol and elimination of nuclear DNA fragments to avoid activation of DNA damage responses (Dellar et al., 2022). RNAs in EVs can be useful for liquid biopsy approaches to identifying, diagnosing or tracking the progression of a certain disease (Chen et al., 2023; Yokoi et al., 2017; Dogra et al., 2020) or targeted therapeutics (Chen et al., 2018; Yokoi et al., 2017; Kamerkar et al., 2017). Additionally, the functionality of RNAs in EVs has been studied which shows that small EVs can induce changes in cellular behaviour via mRNA, miRNA and protein cargo exchange (Valadi et al., 2007; Ratajczak et al., 2006). For example, mice were exposed to 10 µg of total small EV protein as a method of determining an appropriate dose of small EVs (noted as ‘exosomes’ in their work) – the concentration of RNA within this dose was not determined prior to injection, bringing into question the functionality of RNA in EVs for therapeutic use (Hoshino et al., 2015). A standardized method of determining the amount of RNA in EVs is necessary for the continued use of small EV‐RNA in a therapeutic setting. Additional information on EVs for therapeutic use is highlighted in Section 7.

## 4 EPIGENOMICS AND EPI‐TRANSCRIPTOMICS OF EVS

The transfer of RNA, DNA and/or protein cargoes between EVPs from host cells to other cell types has been studied to understand their roles in intercellular communication and epigenetic changes to organisms (Camussi et al., 2011). EVs derived from murine embryonic stem cells have been shown to reprogrammme adult hematopoietic stem/progenitor cells through epigenetic reprogramming (Ratajczak et al., 2006). Multiple observations of EVs inducing epigenetic changes through the delivery of mRNA and miRNA as detailed in the review by Camussi et al. show that the regulation of protein translation and gene expression in neighbouring cells is potentially mediated by EVPs (Camussi et al., 2011).

EVPs have also been utilized as delivery vehicles for gene‐editing CRISPR/Cas tools, as they have high biocompatibility, safety, molecular engineering opportunities, and can cross biological barriers (Kostyushev et al., 2020). Further research is necessary to understand the loading efficiency and delivery of specific cargoes from EVPs, however, they remain an exciting solution to CRISPR/Cas delivery due to their ability to escape endolysosomal pathways, shield their cargoes from aggressive environmental conditions and the immune system and their simple and scalable engineering (Kostyushev et al., 2020). Additionally, EVPs have been studied for their potential in mediating soma to germline information transfer, inducing a transgenerational epigenomic modification (Sharma, 2014). Comprehensive bioinformatic studies have shown that EVs carrying ncRNA, miRNA and mRNAs can induce epigenetic inheritance in mammals (Sharma, 2014). Indeed, the role of the cargoes within EVPs and their impact on host cells through intercellular communication, epigenomic alterations and genetic engineering remain unknown. Future studies on how RNA, DNA and protein from EVPs interact are necessary to further understand their importance in human biology.

## 5 TRANSCRIPTOMICS TECHNOLOGIES FOR EVPS

The ability to analyse an organism's total transcriptome in a given tissue at homoeostasis, or after exposure to certain conditions gives a snapshot into how genes are regulated or give function to previously unannotated genes (Von Felden et al., 2022; Lowe et al., 2017). An organism's transcriptome refers to the sum of all its RNA transcripts, including coding and non‐coding RNAs, which are necessary for cellular function (Padilla et al., 2023). Analyses of the human transcriptome began in 1991, with the sequencing of 609 mRNA sequences from the human brain (Adams et al., 1991). Since then, there have been many technological advances which have allowed EVPs to be sequenced using both long‐read and short‐read technologies (Wang et al., 2021; Padilla et al., 2023; Huang et al., 2020). Herein, we discuss the transcriptomics technologies of EVPs.

### 5.1 Long‐read sequencing of EVs

Long‐read human genome sequencing generates continuous reads ranging from 10 kilobases to several megabases from native DNA (Logsdon et al., 2020). Currently, two main technologies are in use, Pacific Biosciences (PacBio) and Oxford Nanopore Technologies (ONT), which perform using slightly different chemistries and sequence detection methods and can produce reads from highly repetitive regions of the genome with accuracy (Logsdon et al., 2020). Few studies have investigated the RNA transcriptome of EVs using long‐read sequencing technologies. In one such study, poly‐A priming was utilized to enable a full‐length transcript generation through reverse‐transcription of poly‐A positive material (Padilla et al., 2023). The results from this study discovered mainly mRNA, pseudogenes and lncRNA from EVs generated from human K562 cells (Padilla et al., 2023). Another study utilizing long‐read transcriptomics also found a majority of mRNA reads, as well as circRNA and lncRNA generated from peripheral plasma samples of pregnant females (Huang et al., 2020).

In 2015, the first nanopore sequencing device, MinION, was released by ONT (Wang et al., 2021). The release of this technology allowed for an increase in sequencing, data and analyses of DNA and RNA from biologics. There are two options when sequencing RNA with ONT's methods; special library preparation is necessary in which the 3′ end and adaptor of native RNA are ligated without reverse transcription, or cDNA is synthesized to obtain an RNA–cDNA duplex with ligation to the adaptor (Wang et al., 2021). In either case, only RNA is sequenced and accuracy is reported to be ∼85% (Wang et al., 2021). Continued advances in nanopore technology have allowed for the sequencing of technical and low‐abundance samples, such as EVPs (Srinivasan et al., 2019).

### 5.2 Short‐ and long‐read sequencing

Short‐read sequencing is the most used form of sequencing technology in which the genome is broken down into small fragments prior to sequencing, typically between 50 and 300 nt (Logsdon et al., 2020). Short‐read sequencing is typically a lower cost and a higher accuracy option compared to long‐read sequencing (Quail et al., 2012; Korotkevich et al., 2021). However, limitations to short‐read sequencing include difficulty in accurately reporting on pseudogenes or repetitive regions, methylation signatures are not captured, and large copy number and structural variants are not usually detected (Lowe et al., 2017; Padilla et al., 2023).

Currently, the main short‐read sequencing technologies are offered by Illumina and long read by PacBio. PacBio is capable of 10,000 bp reads with 87% single‐read accuracy, whereas Illumina provides 50–300‐bp reads and has a 99.9% single‐read accuracy rating. While Illumina has been considered the gold standard in sequencing technologies, there are major shortcomings to note (Quail et al., 2012; Bolger et al., 2014). First, their short‐reads of 300 bp or less are considered to be too short to detect more than 70% of the human genome structural variation, and over 15% of the genome remains inaccessible to Illumina technologies due to repeat content or atypical GC content (Logsdon et al., 2020). PacBio uses a topologically circular DNA molecule template which allows longer reads (Logsdon et al., 2020). However, adapting long‐read technologies to EV‐RNAs represents a self‐contained technical challenge due to the RNA length heterogeneity inherent to EVs.

### 5.3 Single‐EV transcriptomics

Recently, single‐cell sequencing transcriptomics has been used to analyse the profile of single‐EVPs (Tesovnik et al., 2021; Veziroglu & Mias, 2020). In order to navigate low‐RNA concentrations, single‐cell technologies utilize a modified cDNA amplification protocol to generate libraries sufficient to capture the genome of a single cell with high accuracy (Tang et al., 2009). In 2022, Luo et al. was the first group to utilize sing‐cell sequencing technology to investigate the transcriptomic features in a single EVP (Luo et al., 2022). In this study, EVPs ranging from 100 to 1000 nm in diameter were isolated by successive differential centrifugation and adjusted barcoding methods (Luo et al., 2022). Their results indicated that 2088 and 3935 EVPs from two replicates of K562 cells and 1603 EVPs from MSC cells were captured in their single EV‐RNA transcriptomics experiment. From both cell types, the median number of genes per EV was ∼43 (Luo et al., 2022). Analysis of the top 500 most highly expressed genes indicated that ribosomal genes, mitochondrial genes and haemoglobin genes were present within the EVPs (Luo et al., 2022). More recently, there have been additional attempts to understand EVPs at the single‐particle level, including an analysis of single EVPs produced from malignant breast cancer (Zhang et al., 2023) and cardiomyocyte proteostasis (Schoger et al., 2023).

## 6 ANALYSIS OF EVP TRANSCRIPTOMICS

### 6.1 EVP‐RNA databases

The current online resources specific to EVP‐derived RNA species are summarized in Table 2. These databases compile EVP‐derived RNA resources from various sources, categorizing them based on available information specifically related to isolation source, isolation method, type of EVP population and RNA species. These databases serve various purposes. For instance, ExoBCD focuses on markers for breast cancer, indicating its disease‐specific utility (Wang et al., 2021). Others, like ExoCarta (Keerthikumar et al., 2016) and miREV (Hildebrandt et al., 2021), are dedicated to term ‘exosomes’, while exoRBase 2.0, CMEP (Li et al., 2019), EVpedia (Kim et al., 2015) and ExoBCD (Wang et al., 2021) cater to a broader category of EVPs. Some databases are tailored to a single RNA class, such as EVmiRNA (Liu et al., 2019), CMEP (Li et al., 2019) and miREV (Hildebrandt et al., 2021) which are used for miRNAs, whereas platforms like ExoCarta (Keerthikumar et al., 2016), exRNA Atla (Murillo et al., 2019) and EV Atlas (Bolger et al., 2014; Langmead et al., 2009) offer a comprehensive range of RNA classes. Although these expanding resources are invaluable, they currently lack consistent curation. For instance, miR‐1202 and miR‐1246, identified as miRNAs in miRBase, are in fact snoRNA and a foetal bovine serum contaminant, respectively (Sakha et al., 2016; Alexander et al., 2022; Tosar et al., 2017). Such mis‐annotations may stem from variations in curation methods utilized by different databases. Consequently, a well‐curated and standardized resource for EVP‐RNAs would significantly benefit the field.

**TABLE 2:** Comprehensive summary of current online resources specific to EVP‐derived RNA species.

Database	Reported EV subtype	RNA class	References
ExoCarta	Exosomes	mRNA, miRNA, rRNA, tRNA, snoRNA, snRNA, lncRNA, lincRNA, ncRNA	Keerthikumar et al. (2016)
exRNA Atlas	Extracellular vesicles, exosomes, HDL complex, lipoproteins, total cell‐free biofluid RNA	miRNA, piRNA, tRNA, Y RNA, lincRNA, snoRNA, snRNA	Murillo et al. (2019)
Vesiclepedia	Apoptotic bodies, ectosomes, exosomes, extracellular vesicles, large dense core vesicles, membrane blebs, microparticles, microvesicles, oncosomes, outer membrane vesicles, prostasomes	mRNA, miRNA	Pathan et al. (2019)
EVmiRNA	Exosomes, microvesicles	miRNA	Liu et al. (2019)
EV Atlas	Small/Large EV	miRNA, snoRNA, piRNA, snRNA, rRNA, tRNA, Y RNA	Bolger et al. (2014), Langmead et al. (2009)
miRandola	Argonaute2 protein, exosomes, microvesicle, microparticle, membrane vesicle, high‐density lipoprotein	miRNAs, circRNAs, lncRNAs	Russo et al. (2018)
exoRBase 2.0	Exosomes	mRNA, circRNAs, lncRNAs	
EVpedia	Extracellular vesicles	mRNA, miRNA	Kim et al. (2015)
CMEP	Exosome, microvesicles	Circulating miRNAs	Li et al. (2019)
ExoBCD	Exosomes	mRNAs, miRNAs, lncRNAs	Wang et al. (2021)
Human Biofluid RNA Atlas		mRNA, circRNA and miRNA	Hulstaert et al. (2020)
miREV	Exosomes	miRNA	Hildebrandt et al. (2021)

### 6.2 Bioinformatics pipelines for EVP transcriptomics

Unlike the transcriptomic analysis of cellular RNA, for EVP‐derived RNA, standard bioinformatics pipelines often need to be adapted or customized to account for the heterogeneous class of EV‐derived RNA. Similarly, like current RNA databases, the pipeline for processing EVP‐RNA also serves a specific purpose, depending on the RNA class and size. A bioinformatics pipeline exceRpt (Rozowsky et al., 2019) is designed specifically for short RNAs. While several small RNA‐seq analysis pipelines are available (Bezuglov et al., 2023), they are typically limited to certain RNA species and often utilize different RNA databases, posing challenges for cross‐study comparisons. Annotation of genomic regions without using a reference is rarer due to their limited interpretability; however, several groups have demonstrated the capacity of *de novo* transcriptome discovery and assembly to identify and quantify biologically relevant transcripts. Such approaches include Wajnberg et al. using a find‐then‐annotate approach to analyse unannotated expression RNAs (Wajnberg et al., 2023) and Felden et al. using a read clustering approach to predict early hepatocellular carcinoma (Von Felden et al., 2022). The few pipelines that can analyse more than one RNA type are still inefficient for total RNA analysis. Therefore, there is a need for a standardized and customized pipeline capable of analysing a broad spectrum of small RNA species of varying sizes, including annotated and unannotated genomic regions, to facilitate consistent and comparable research outcomes. To achieve this, the choice of alignment tools and parameters might require adjustments for the uneven nature of EV‐RNA, which includes a mixture of small RNAs. This is followed by utilizing appropriate normalization and quantification techniques, given the variability in EVP‐RNA yield and differences in EV production and RNA packaging across conditions.

## 7 LIQUID BIOPSY AND TARGETED EVP THERAPIES

Liquid biopsy is a minimally invasive tool for early diagnosis, treatment selection and long‐term follow‐up of cancer and numerous diseases that can be performed procedures, and frequently uses the patient's body fluids such as urine or blood (Chen et al., 2022; Gaglani et al., 2021). Unlike tissue biopsy, liquid biopsy is less susceptible to spatial heterogeneity and can provide information on metastatic tumour tissue (Parikh et al., 2019). Although liquid biopsies typically analyse circulating tumour cells (CTCs), circulating tumour DNA (ctDNA), cell‐free RNA (cfRNA) and miRNA in body fluids such as blood and urine (Ye et al., 2019), EVPs have recently attracted much attention (Kalluri & LeBleu, 2020; Vasconcelos et al., 2019; Boivin et al., 2019; Kim et al., 2017; Xu et al., 2022; Yokoi et al., 2017; Li et al., 2017). Liquid biopsies have been developed to examine miRNAs and mRNAs contained in small EVs, such as exosomes, and there are reports that the number of specific miRNAs has increased in cancer patients (Jin et al., 2017; Madhavan et al., 2015). There is also a report of an omics analysis that used exosomal RNA in plasma to stratify the effect of preoperative chemotherapy in cancer patients (Guo et al., 2022). Furthermore, RNA‐seq in tumour culture supernatants from prostate cancer patients, as well as blood and urine EVs before and after resection found differential expression of reactive oxygen species, p53 pathway, inflammatory/cytokine, oncogenes and tumour suppressor genes in EV nanosatellites (Chen et al., 2022). This comparison of RNA in the EVs before and after treatment demonstrated the potential of liquid biopsies for non‐invasive monitoring. It also demonstrates that EVs unannotated RNAs (small RNA clusters) may play important new roles in the regulation of gene expression, post‐transcriptional and post‐translational mechanisms (Dogra et al., 2020). However, there is a large variability in results due to the methods used to recover small EVs (Royo et al., 2020). Thus, the challenges in developing small EV‐based liquid biopsy, coupled with the problem of high heterogeneity (Raposo & Stoorvogel, 2013), require further research for the practical application of exosome‐based liquid biopsy in the clinic (Yu et al., 2022; Kowal et al., 2016). Additionally, EVPs are used in differing targeted therapies involving both human and murine research models.

For EVP‐based therapies, both human and murine in vitro and in vivo EV therapies are being researched. Current human trials include EV therapies used in anti‐inflammatory (Warnecke et al., 2021; Schultz et al., 2021; Abdelgawad et al., 2021), anti‐tumour (Weng et al., 2021; Nicodemou, 2023; Shan et al., 2024; Lee et al., 2013), immunomodulatory (Escudier et al., 2005; Besse et al., 2016; Narita et al., 2015) and drug delivery (Dai et al., 2008) summarized in Table 3. Notable examples include reports that mesenchymal stem cells (MSCs) derived EVs can suppress inflammation caused by cochlear implantation and are useful in neurodegenerative diseases due to their own anti‐inflammatory properties (Warnecke et al., 2021; Hoecke et al., 2021; Cone et al., 2021).

**TABLE 3:** A summary of EVP therapies, including their effect, source and targeted disease.

Effect	EV source	Research Model	Target diseases and treatment methods	References
Anti‐inflammatory	Mesenchymal stem cells	Human Study	Reduce inflammation by cochlear implantation	Warnecke et al. (2021)
	Mesenchymal stem cells	Murine Studies	Niemann‐Pick Type C disease, Alzheimer's Disease	Hoecke et al. (2021), Cone et al. (2021)
	Mesenchymal stem cells	Murine and Human Study	Acute lung injury	Lee et al. (2019), Hao et al. (2019)
	Mesenchymal stem cells	Human Study	COVID‐19	Schultz et al. (2021), Abdelgawad et al. (2021)
Anti‐tumour	Mesenchymal stem cells	Murine and Human Study	Cancer therapy (suppress angiogenesis)	Weng et al. (2021), Nicodemou. (2023), Shan et al. (2024), Lee et al. (2013)
Immunomodulatory	Mesenchymal stem cells	Human Study	Inflammatory disease	Seo et al. (2019)
	Dendritic cells	Human Study	Cancer vaccine	Escudier et al. (2005), Besse et al. (2016), Narita et al. (2015)
	CD8^+^ T cells	Murine Study	Cancer therapy	Seo et al. (2018)
Drug delivery	Adeno‐associated virus‐producing cells	Murine Study	Gene delivery for heart failure treatment	Li et al. (2023)
	Mesenchymal stem cells	Murine Studies, Clinical Trials	siRNA delivery for cancer therapy	Cheng and Hill, 2022), Kamerkar et al. (2017), Mendt et al. (2018)
	Dendritic cells	Murine Study	siRNA delivery for Alzheimer's disease	Alvarez‐Erviti et al. (2011)
	Ascites	Human Study	GM‐CSF delivery for cancer therapy	Dai et al. (2008)
	Macrophage	Murine Study	Paclitaxel delivery for cancer therapy	Kim et al. (2016)
Suppression of EVs secretion	Cancer cells	Murine Study	Cancer therapy (anti‐tumour immunity)	Poggio et al. (2019), Guan et al. (2022)
	Hepatocellular cell carcinoma cells	Murine Study	Cancer therapy	Hu et al. (2023)
	Prostate cancer cells	Murine Study	Cancer therapy	Urabe et al. (2020)

Next, regarding immunomodulatory effects, immune cell‐derived EVs contain a variety of molecules that activate or suppress immunity and are involved in both innate and acquired immunity. Among them, EVPs derived from dendritic cells (DCs), one of the antigen‐presenting cells, express MHC‐I and MHC‐II and are known to be involved in antigen presentation and to stimulate tumour‐specific immune responses (Zitvogel et al., 1998; Lynch et al., 2009; Pitt et al., 2016). In EVPs derived from cancer cells, PD‐L1+ small EVs have been reported to be associated with the therapeutic efficacy of immune checkpoint inhibitors and prognosis because PD‐L1 is expressed on the surface of small EVs released from cancer cells and causes tumour growth by suppressing CD8+ T cell function (Theodoraki et al., 2018). A remaining challenge in the use of EVPs as therapeutics is the risk of infection caused by intravenous administration of small EVs, therefore, there is an urgent need to establish guidelines for the therapeutic application of EVPs based on efficacy and quality control of EVPs.

## 8 CURRENT CHALLENGES, POSSIBLE SOLUTIONS, AND RECOMMENDATIONS

In the last decade, ISEV has made tremendous progress with EV research and also identified critical limitations with EV‐RNA investigation and missing opportunities (Théry et al., 2018; Welsh et al., 2024). For instance, major gaps in knowledge in EV‐RNA isolation, library prep, sequencing and bioinformatics analyses (suboptimal alignment, lack of multimapping quality control, RNA contaminants, missing RNA cargo types and lack of standardized analytical tools) have been identified with current standard approaches (Murillo et al., 2019; Welsh et al., 2024). These limitations reduced the discovery capabilities of liquid‐biopsy technologies among the international scientific community (Welsh et al., 2024). We also now understand that vesicle heterogeneity plays a key role in EV transcriptome composition (Lässer et al., 2011; Crescitelli et al., 2021; Karimi et al., 2022). Therefore, there is a critical need to overcome these limitations and provide new open‐access algorithms to advance our understanding of EVP transcriptomes. At present, understanding the small RNA landscape of EVPs is a major bottleneck when it comes to delineating their role in disease, specifically for accurate diagnostics. Here, we highlight current challenges, discuss possible solutions and present recommendations for robust and reproducible analyses of EVP‐associated small RNAs.

### 8.1 Challenge 1: Small amounts of EV‐RNA recovered from biofluids may lead to a lack of reproducibility

A recent worldwide survey (Royo et al., 2020) reported that two of the most popular human biofluids used for EV isolation are plasma and serum. A major challenge involves small‐volume inputs from plasma and serum that are acquired for EVP isolation methods. For example, ∼2‐mL aliquot of serum may result in ∼200 pg/µL of RNA (Chen et al., 2022), thus resulting in a small sampling of RNA molecules and a lack of reproducibility. Furthermore, different RNA isolation methods for small RNA offer several pros and cons providing different yields and lengths of RNA. EV isolation technology can also lower the EV‐RNA yield. For instance, SEC is a rapidly growing tool for EV isolation (Gámez‐Valero et al., 2016); however, SEC dilutes the sample concentration by a factor of ∼10–100 (Böing et al., 2014; Gámez‐Valero et al., 2016). Hence, this process lowers the EV‐RNA recovered from biofluids. Additionally, it should be noted that within isolated EVs, there are low concentrations of RNAs. A study by Chevillet et al. reported that there was less than one miRNA per isolated EV particle (Chevillet et al., 2014). Our group has reported similar findings, where approximately 1.4 × 10^8^ EV particles were necessary via SEC isolation in order to obtain enough RNA for proper bioinformatics pipeline analysis (Soleymani et al., 2023; Chen et al., 2022). Heterogeneity of RNA in EVs is also a problem, as some researchers have noted that there may be a lack of miRNA enrichment in small EVs (Chevillet et al., 2014; Albanese et al., 2021; Balkom et al., 2015), whereas others have suggested high enrichment of tRNAs in small EVs (Chevillet et al., 2014; Nolte‐'t Hoen et al., 2012). Possible solutions and recommendations: Recently reported by Tkach et al. (2022) and others (Liangsupree et al., 2021; Oeyen et al., 2018), a downstream (post‐SEC) benchtop centrifugal ultrafiltration/concentration by molecular weight cut‐off (MWCO) has provided higher EV yields. We recommend an integration of ultrafiltration with specific MWCO to purify and concentrate small EVs. As discussed in Table 1, ultrafiltration by MWCO can further enhance EV purity, given that VLDLs, HDL and small EVs differ in MW by a factor of ∼10–100 kDa, respectively (Table 1). Importantly, we do not recommend using RNA concentration kits for EV‐RNA because most kits are designed for cellular RNAs, and some may bias RNA enrichment by their lengths and specificity.

### 8.2 Challenge 2: EV‐RNA quality controls are not well established

The current RNA quality control standards are developed based on cellular RNA analyses that historically focused on well‐established annotated genomic regions (e.g. miRNA, mRNA, rRNA). However, since small EVs predominantly carry small RNA molecules, it is apparent that EV‐specific RNA requires a novel approach for a better understanding of their molecular cargo (Valadi et al., 2007; Bellingham et al., 2012; Murillo et al., 2019). For instance, the well‐established quality control metric ‘RNA integrity number’ (RIN) – which evaluates the area under the curve of large ribosomal RNA (18S and 28S) separated via electrophoresis, is unsuitable for EV‐associated small RNA (Valadi et al., 2007; Schroeder et al., 2006). Furthermore, nanodrop (Thermo Scientific) and qubit (Invitrogen) can provide qualitative information on EV‐associated small RNA. Possible solutions and recommendations: It is important to note that the EV‐RNA QC protocols may fail and this does not always indicate that the EV‐RNA is degraded (see Section 5.1, nanopore *long‐read sequencing*). We recommend assessing the precise length of RNA of EVs, through capillary electrophoresis using two separate analyses kits: Pico and small RNA kits for bioanalyser (Crescitelli et al., 2013). The size distribution and quantity of genetic materials extracted from the EVs should be evaluated using an electropherogram before and after cDNA library preparation (discussed in challenge 3) (Tesovnik et al., 2021). The electropherogram curve for EV‐RNA samples tends to carry relatively shorter sequences of up to 200 nucleotides with no distinctive peak for 18S and 28S ribosomal RNA; the presence of 18S or 28S peak could denote contamination from cell debris during the isolation process (Tesovnik et al., 2021; Tang et al., 2017). Likewise, conventional RNA and DNA integrity numbers devised to evaluate genetic material qualities are less reflective of EV‐RNA quality given their short sequence length (Tesovnik et al., 2021).

### 8.3 Challenge 3: EV‐RNA‐seq protocols (from cDNA prep to sequencing) are not well‐established

To distinguish the technical variance from the biological differences, it is essential to establish references and processing controls for effective RNA quantification prior to downstream computational processing (Tesovnik et al., 2021). Library prep methods comparison, RNA input quantity (e.g. amount of starting material) remains an open question. Currently, several small RNA library prep kits (Takara bio, true seq, NEBNext etc.) are used for EV‐RNA library prep. It is found that the choice of library preparation impacts the miRNA sequences detected by RNAseq (Srinivasan et al., 2019). Possible solution and recommendations: Total small RNA sequencing and total RNA sequencing are necessary to capture the complete landscape of RNA subtypes found within EVPs. Studying only the miRNA content may be ignoring up to 70%–90% of the total EVP cargo (Chen et al., 2022). Furthermore, studies have verified that the addition of ERCC RNA or DNA spike‐in to all samples could allow for effective evaluation of the trueness of nucleotide quantification during the library preparation process (Tesovnik et al., 2021; Hulstaert et al., 2020). This is especially important for EVP samples that tend to have low‐input materials and varying concentrations of EVPs and are critical for normalizing sequencing reads or determining the original RNA concentration of the samples (Everaert et al., 2019; Hulstaert et al., 2020). Everaert et al. provided an example of determining the RNA recovery rate of their library preparation via the correlation between expected and observed spike‐in genetic materials; a high correlation from the spike‐in would suggest outstanding RNA recovery for all samples from the same batch processed using the same library preparation and sequencing workflow (Everaert et al., 2019).

### 8.4 Challenge 4: EV‐RNA alignment and quantification approaches are not well standardized

The existing small RNAseq alignment methods are largely developed for cellular small RNAs (Eldh et al., 2012; Srinivasan et al., 2019), which are inherently different RNA subtypes than those found in EVs (Chen et al., 2022). Small RNA sequences suffer from a multimapping read percentage due to their short length aligning in multiple genomic regions (Ziemann et al., 2016). The short read alignment problem is well established in bioinformatics and has led to the development of diverse algorithms to carefully weight multimapping reads in the vicinity of uniquely mapping reads and thereby increase specificity and sensitivity (Ziemann et al., 2016; Handzlik et al., 2020; Deschamps‐Francoeur et al., 2020). However, none of these methods have been tested in EV‐RNAs or fragmented RNA. More importantly, short‐length RNAs are often confused with degraded and fragmented RNAs. This results in low number of alignments or low mapping quality compared to cellular RNA, which is misinterpreted as failed sequencing. Further, sequencing facilities often use these profiles to quality control cellular RNA and misidentify these patterns as arising due to fragmentation or degradation leading to the discarding of short‐length EV‐RNAs. Thus, new quality control approaches that are tailored for short‐length RNAs are critically needed. Finally, available databases may differ not only in their curation methods, but also in the variety of aligner tools and methodologies used for data preprocessing. For example, the exRNA Atlas employs the exceRpt (Rozowsky et al., 2019) aligner, while the HumanBiofluid RNA Atlas (Hulstaert et al., 2020) utilizes STAR and Bowtie aligners for mapping reads to the reference genome. Benchmarking studies, such as the one cited (Bezuglov et al., 2023), have demonstrated that the choice of aligner can significantly affect the recovery of reads from different RNA classes. Therefore, comprehensive integration and comparison of these resources are essential to address discrepancies between studies utilizing specific databases. Possible solutions and recommendations: To address these challenges, there is an urgent need to develop novel approaches and extensively benchmark these technologies tailoring them to EV transcriptomes. Potential solutions to this problem include using short read‐specific aligners such as bowtie or BWA, which can improve the alignment rate of short sequences under 50 bp (Lu et al., 2018; Bezuglov et al., 2023). Similar methodology is used recently by (Dogra et al. 2024) to identify EV‐associated ‘dark matter’ RNA or EV‐associated unannotated genomic regions (EV‐UGRs). We recommend that using positive and negative controls for sequencing such as human reference UHRR, non‐human miRNA or environmental controls can help understand better when the low mapping is due to low‐quality RNA or a lack of material and quantify true EV‐RNAs. Similarly, We recommend several databases to verify the presence of common contaminants or known bacterial specifies, such as UniVec (https://www.ncbi.nlm.nih.gov/tools/vecscreen/univec/) or microbial genomes (https://www.ncbi.nlm.nih.gov/genome/microbes/), respectively. These databases can be used to further filter or characterize the EV transcriptome. Yet, new experimental and computational approaches are needed to discover, characterize and validate previously ignored regions in the genome as possible biomarkers of disease. Such standardized bioinformatics pipelines need to be established, tested and proposed by MISEV and other EV communities as collaborative efforts.

### 8.5 Challenge 5: EV‐RNA differential expression, gene set enrichment and signatures require careful considerations

EV transcriptomics analysis offers crucial insights into cellular functions and disease mechanisms by examining RNA content associated with EVs (Bellingham et al., 2012). This approach reveals the interactions between genes and pathways, enhancing our understanding of health and disease at the genetic and molecular levels (Subramaniana et al., 2005). It aids in identifying new diagnostic markers and potential therapeutic targets (Subramaniana et al., 2005). However, analysing the EVP transcriptome computationally is more challenging than traditional cellular transcriptomics (Padilla et al., 2023). The diversity of EVP isolation and library preparation methods, along with the heterogeneity of RNA types in EVP populations, presents unique challenges (Turchinovich et al., 2019). These factors, combined with the choice of sequence aligner and annotation database for various EVP RNA types, can significantly affect EVP RNA mapping, size distribution and abundance (Everaert et al., 2019; Mugoni et al., 2022). Consequently, these variations influence the comparison of transcriptomic analyses across different studies unless specific details – such as the isolation source, method, type of EVP population and RNA species – are explicitly stated. However, recent developments in online EVP‐specific resources (discussed in Section 6), databases and pipelines for processing EVP‐derived RNA‐seq data have begun to alleviate these challenges. Possible solutions and recommendations: When compared with their cell of origin, EVs carry distinct proteo‐transcriptome (Chen et al., 2022). However, a comparison of cellular and EV‐RNA must be conducted with caution. For instance, EVs carry relatively smaller RNAs than their cell of origin. The lower detection limits of EV‐RNA could lead to biased results and their interpretation is under intense debate currently (Chen et al., 2022). The recent studies (Chen et al., 2022; Chen et al., 2023; Bezuglov et al., 2023), using benchmarking approaches, recommend tools for small RNA‐seq analysis, including trimming, filtering, mapping, transcript abundance quantification and differential expression analysis (Bezuglov et al., 2023). A general recommendation is to perform careful data cleaning for non‐species specific transcriptomes and contaminants, as well as performing a full transcriptome analysis not limited to mRNAs and using reference mapping and reference agnostic approaches such as find‐then‐annotate, *de novo* discovery or read clustering to maximize the identification of EV‐RNAs (Von Felden et al., 2022; Wajnberg et al., 2023).

### 8.6 Challenge 6: Reproducible PCR validation of genes identified from EV‐RNA sequencing

Owing to its powerful impact and simplicity, RT‐qPCR validation of EV‐RNAs (identified through RNA sequencing) is highly desirable. However, the RT‐qPCR validation of identified EV‐RNA may not produce the expected results if only a part of gene is present in EVs. Possible solutions and recommendations: EVs have been shown to carry intact and fragmented RNA (Kim et al., 2017; Prieto‐Vila et al., 2021). Hence, during the genomic analyses, only specific genetic loci of a gene may show high expression. We recommend designing PCR primers by carefully identifying the genomic region (genetic loci) of RNA that exhibits the highest expression in EVs. We do not recommend PCR primer design by cumulative total expression of a given gene.

## 9 CONCLUSION AND FUTURE DIRECTIONS

In this article, we provide clarity on the transcriptomics landscape of EVs through an in‐depth analysis of EVPs, their small RNAs landscape and the use of several transcriptomics technologies. Overall, we highlighted comprehensive online resources, current bioinformatics pipelines specific to EVP‐derived RNA species and clinical applications of EVPs. Finally, we outlined challenges and recommendations for continued EVP‐transcriptomic analyses, aiming to propel the field forward and enhance the accessibility and comprehensiveness of published works for cross‐comparison. Such challenges include small amounts of RNA from isolation methods, and a lack of appropriate bioinformatics pipelines, quality controls and sequencing protocols. Detailed descriptions of the current state of EVP transcriptomics will lead to a better understanding of how the RNA cargo of EVPs can be used in modern and targeted diagnostics and therapeutics. The success of the Human Genome Project, cancer moonshot and Genotype‐Tissue Expression (GTEx) have revolutionized our understanding of the gene expression in innumerable diseases (Veziroglu & Mias, 2020; Lowe et al., 2017; Adams et al., 1991; Pavlova et al., 2022). In this context, there is an urgent need for cross‐institutional, disease‐independent, interdisciplinary launch of an EV‐RNA ‘moonshot’ project.

## AUTHOR CONTRIBUTIONS

Navneet Dogra conceived the study. Rebecca T. Miceli, Yohei Nose, Swapnil Tichkule, Edgar Gonzalez‐Kozlova, Tzu‐Yi Chen and Navneet Dogra wrote the manuscript. Rebecca T. Miceli, Yohei Nose, Swapnil Tichkule, Briana Brown, John F. Fullard, Marilyn D. Saulsbury, Simon O. Heyliger, Sacha Gnjatic, Natasha Kyprianou, Carlos Cordon‐Cardo, Susmita Sahoo, Emanuela Taioli, Panos Roussos, Gustavo Stolovitzky, Edgar Gonzalez‐Kozlova, Tzu‐Yi Chen and Navneet Dogra reviewed, edited, read and approved the manuscript and guided the interpretation of the manuscript.

## CONFLICT OF INTEREST STATEMENT

S.G. reports past consultancy or advisory roles for Taiho Pharmaceuticals; research funding from Regeneron Pharmaceuticals, Boehringer Ingelheim, Bristol Myers Squibb, Celgene, Genentech, EMD Serono, Pfizer and Takeda, unrelated to the current work. All other authors declare that the research was conducted in the absence of any commercial or financial relationships that could be construed as a potential conflict of interest. N.D. and G.S. are awarded a patent entitled ‘Exosome vessels for delivery of molecular cargo’, US Patent 11,766,484.
